# Effects of Microbial Transglutaminase on Technological, Rheological, and Microstructural Indicators of Minced Meat with the Addition of Plant Raw Materials

**DOI:** 10.1155/2020/8869401

**Published:** 2020-12-19

**Authors:** Oksana Zinina, Svetlana Merenkova, Damir Galimov, Eleonora Okuskhanova, Maksim Rebezov, Mars Khayrullin, Olga Anichkina

**Affiliations:** ^1^South Ural State University (National Research University), 76 Lenin Avenue, Chelyabinsk 454080, Russia; ^2^Shakarim State University of Semey, 4 Phizculturnay Str. Semey 071410, Kazakhstan; ^3^V.M. Gorbatov Federal Research Center for Food Systems of Russian Academy of Sciences, 26, Talalikhina Str., Moscow 109316, Russia; ^4^K G Razumovsky Moscow State University of Technologies and Management (The First Cossack University), 73 Zemlyanoy Val, Moscow 109004, Russia

## Abstract

The aim of the study was to analyse the effects of transglutaminase on the physicochemical, technological, rheological, and microstructural indicators of minced meat with the addition of plant raw materials. The formulations of minced meat from beef trimming, hemp protein, and flax flour were optimized in terms of biological value and optimal content of essential amino acids. The addition of plant components in amounts greater than 18% caused an increase in the content of protein, fat, and ash in the minced samples. The rheological properties of minced meat samples without enzyme treatment changed depending on the proportion of plant raw materials. When the content of the flax flour was increased, the minimum ultimate shear stress and viscosity were observed, while the maximum values for these indicators were achieved in samples containing about 15% hemp protein, as well as in samples without plant additives. When adding transglutaminase to the formulation, increases in the ultimate shear stress and viscosity were proven for all combined minced samples. The combined minces, containing flax flour, had a more plastic structure and the lowest modulus of elasticity, while minces including 14% hemp protein or more than 87% meat components were identical to the control samples in terms of deformation and elasticity. In enzyme-treated minces, the plasticity of the samples reduced while density and elasticity increased. Transglutaminase treatment contributed to the formation of optimal technological properties of combined minces. Microstructural analysis showed the intermolecular bonds between protein particles in combined minced samples with the addition of enzymes. The research results have demonstrated the effectiveness of using transglutaminase in the composition of combined minced meat for the formation of a homogeneous and dense system with the necessary technological and rheological properties.

## 1. Introduction

One of the important directions by which to ensure public health is to expand the range of products for a healthy diet, which are characterized by high nutritional and biological value and include essential components in balanced ratios. A balanced diet provides full vitality and helps to prevent pathological disorders and human diseases. It is important to study approaches that use the application of plant raw materials with proven functional properties in the technology of combined products, which allows one to modify the composition and adjust the technological properties of food accordingly.

Due to the current deficit of animal protein in the world, the population's needs are being met using other sources, mainly of plant origin [1].

The increased requirement for protein sources and, at the same time, the need to ensure a rational diet have led to the emergence and development of a new direction in food production, namely, one of obtaining combined foods based on the potential resources of dietary proteins of both animal and plant origins. The combination of meat- and vegetable-based raw materials provides high nutritional value of processed products, increases the variability of formulations, leads to a homogeneous distribution of ingredients, minimizes losses during heat treatment, and ultimately contributes to the creation of products of stable quality.

The introduction of plant ingredients into minced meat is one of the ways in which one can obtain high-quality meat products with adjustable compositions and properties. However, it has mainly been proposed to introduce plant protein sources such as soy, chickpea, wheat, lentils, and peas into meat systems [2–5], even though, from the point of view of food chemistry, promising sources of protein include such ingredients as flax and hemp seeds.

Flaxseed is an important source of plant protein, which comprises 18 to 22 g/100 g of seed weight [6]. Flaxseed protein is composed of a salt-soluble and a water-soluble protein fraction. Flaxseed protein poses potential health benefits to populations due to high aspartic acid, glutamic acid, leucine, and arginine content and also its balanced amino acid profile [7]. Flaxseed proteins have a higher capacity to absorb water and oil than soybean protein isolates [8].

Hemp seeds have begun to be used in various foods with high nutritional value. Hemp protein showed a high degree of digestibility and hypoallergenic properties [9]. Hemp seed contains *ω*-3 and *ω*-6 essential fatty acids in optimal ratio and proteins (25 g/100 g dry weight) in which the amino acid profile includes all the essential amino acids [9, 10].

In the traditional technology of minced products, the problem of obtaining a uniform product with homogeneous structure is solved by adding food additives, generally of inorganic origin (for example, phosphates). In the manufacture of combined products containing a significant amount of plant components, the phosphate content might be increased to achieve the necessary texture, which is unacceptable according to sanitary and hygienic standards, especially in healthy food product technology.

One of the approaches to solving the problem may be application the enzyme preparations to obtain a dense, homogeneous structure of the combined product. Transglutaminase (TG) produces inter- and intramolecular cross-linking bonds in the proteins [11]. Lantto et al. [12] noted works in which studies of the effects of TG on proteins of various origins are presented, e.g., about catalysing bonds between meat and soy proteins or between meat, casein, and gluten. Cross-linking proteins containing various essential amino acids improve nutritional value, and therefore, such combined proteins are valuable in food production [13, 14].

The aim of the study was to evaluate the physicochemical, technological, rheological, and microstructural characteristics of the combined minced meat formulations, optimized in terms of biological value, containing beef trimming, flaxseed flour, and hemp protein, with and without the transglutaminase treatment.

## 2. Materials and Methods

### 2.1. Raw Materials and Ingredients

Fresh beef trimming (90/10) (66.34% moisture, 17.40% protein, and 9.26% fat) were obtained from rib and round portions 48 h after slaughter. Beef trimmings were packaged in plastic bags and transported in a refrigerator at 4°C to the laboratory of the Food and Biotechnology Department within 2 h for further processing. The pH of the beef trimmings was determined using a portable pH meter (HANNA HI83141). An electrode was inserted to a depth of 5 ± 1 cm, where the values obtained ranged from 5.56 to 5.62.

Flaxseed flour (9.1% moisture, protein: 39.3% dry matter (DM), fat: 21.1% DM) was produced from the flax seeds of the Racial variety at the Medal enterprise (Chelyabinsk, Russia).

To obtain the flour, flax seeds were heat-treated at 90°C for 3-5 minutes, defatted by pressing, ground in a colloid mill, and sifted through a sieve with a mesh size of 160 microns.

Hemp protein (9.8% moisture, protein: 56.1% DM, fat: 23.4% DM) was produced from the hemp seeds of the Nadezhda variety at the Medal enterprise (Chelyabinsk, Russia).

To obtain the protein, hemp seeds were heat-treated at 70°C for 10 minutes, defatted by pressing, crushed, and sieved several times. A GMSM-120 industrial mill with sieving equipment was used. The resulting hemp protein and flaxseed flour were stored at a temperature of 15-18°C.

Transglutaminase is an enzyme preparation produced by cultivation of *Streptomyces mobaraensis* (BioBond-TG-EB-3; activity: 3.5-6.8 unit/100 mg; Shanghai Kinry Pharmaceutical Co. Ltd.).

### 2.2. Optimization of the Combined Minced Formulation in Terms of Biological Value

#### 2.2.1. Optimization of the Minced Formulation

Beef trimming (90/10), hemp protein, and flaxseed flour were chosen as starting components for the combined minced formulation.

The optimization of the combined minced formulation was carried out according to the essential amino acid index (EAAI), which was calculated using the following formula:

(1) $$\begin{array}{c} \text{EAAI}=\sqrt[{n}]{a_{1}\cdot a_{2}\cdot \cdots a_{n}}, \end{array}$$

where *a*_*n*_ is the ratio of the amount of each essential amino acid in the investigated protein to its amount in the whole egg protein and *n* is the amount of EAA (*n* = 8).

The combined minced formulation was optimized using the SOLVER standard software available in Microsoft Excel (v.2013). The calculation of the formulation consisted of several stages: compiling a data bank and balance equations for the amino acid composition, defining the objective function to optimize formulations, solving the problem using the tool SOLVER, and analysing and selecting a formulation appropriate to the goal. The content of essential amino acids was experimentally detected in the components of the mince via HPLC (high-performance liquid chromatograph) to compile a data bank.

#### 2.2.2. Preparation of Samples for Hydrolysis

Beef trimmings were chopped in a meat grinder (Fimar 32/RS, Unger, Italy) with a plate having 3 mm diameter holes. The hydrolysis of samples of ground beef trimming, hemp protein, and flaxseed flour was carried out according to the method described by Zinina et al. [15]. The amino acid modification was carried out by phenylisothiocyanate solution in isopropanol to obtain phenylthiohydantoins. The concentration of amino acids in the samples was calculated according to the protein content in grams per 100 g of the product. The protein content of the beef trimming, hemp protein, and flaxseed flour samples was determined using the Kjeldahl method.

#### 2.2.3. Determination of Amino Acid Composition

The amino acids were determined on a high-performance liquid chromatograph (Shimadzu LC-20 Prominence, Japan) with a chromatography column of dimensions 25 cm∗4.6 mm SUPELCO C18, 5 *μ*m (USA). Chromatographic analysis was carried out at an eluent flow rate of 1.2 ml/min and a column thermostat temperature of 40°C. The measurement was performed by HPLC on a reverse phase column with fluorimetric and spectrophotometric detectors at wavelengths of 246 and 260 nm. A mixture of 6.0 mM CH_3_COONa solution at pH 5.5 (component A), 1% isopropanol in acetonitrile solution (component B), and 6.0 mM CH_3_COONa solution at pH 4.05 (component C) was used as the mobile phase.

### 2.3. Preparation of Combined Minced Samples

The beef trimming (90/10) was chopped through a grinder (Fimar 32/RS, Unger, Italy) fitted with a plate with 6 mm diameter holes. The minced meat samples were made in accordance with the formulations obtained by optimization, as presented in Table 1.

Hemp protein and flaxseed flour were hydrated in water at a ratio of 1 : 2, respectively. After uniformly mixing all components with water (20% by weight of raw materials), the combined minces were divided into two parts. Transglutaminase (0.2% by weight of raw materials), previously dissolved in water at a ratio of 1 : 10, was added to one part of each of the minces. A similar amount of water was added to the minces without the addition of transglutaminase. The minced samples from beef trimming were taken as the control sample. Then, all the minced samples were kept at 8°C for 2 h to react with the TG. The following designation for samples was introduced: S-C—minced samples produced from beef trimmings; S-C+TG—the minced samples produced from beef trimmings with added TG; S-1, S-2, S-3, and S-4—the combined minced samples produced according to formulations 1, 2, 3, and 4, respectively; S-1+TG, S-2+TG, S-3+TG, and S-4+TG—the combined minced samples produced according to formulations 1, 2, 3, and 4, respectively, with added TG. Thus, a total of 10 formulations were taken, with five minced samples made for each formulation.

### 2.4. Physicochemical Analyses

The chemical analyses of the combined minced samples were conducted in triplicate according to the methods described by AOAC [16]: total nitrogen content was assayed by the Kjeldahl method with nitrogen converted into the equivalent protein content using a factor of 6.25 (methods 992.15 and 992.23); moisture was determined according to method 950.46 B; total fat was determined via the Soxhlet method (methods 920.39 C and 960.39). The ash content was determined via the dry ashing method (method 920.153).

### 2.5. Rheological Measurements

Rheological measurements were conducted using a rotary viscometer (Brookfield R/S SST). The principle of operation is to determine the viscosity and shear stress by measuring the rotation speed of the spindle, which is immersed in the test mince. Viscosity measurement and shear stress range are determined by the spindle speed, the size of the spindle, and the full-scale torque of the calibrated spring. The size of the selected spindle was 30/15, and the full-scale torque was 80%. Viscosity and shear stress values were calculated using a coefficient of 9. Investigations of the rheological properties (ultimate shear stress, effective viscosity) of combined mince were carried out at room temperature (20 ± 1°C).

### 2.6. Texture Measurements

Deformation indicators were conducted using a texture analyser “Structurometer ST2” (LAB, Quality Laboratory, Russia) by compressing it with an indenter “Cylinder Ø36” (duralumin, mass 42.5 g). The analysis of the mechanical tension induced on a cylindrical indenter was carried out under the following operation mode: contact force (Fc = 7 g), strain rate (Vd = 0.5 mm/s), and continuation of the introduction of the indenter until the effort *F*_max_ was equal to 500 g. The total plastic and elastic deformations were determined.

All texture measurements were carried out after sample fermentation for 2 h at a temperature of 8 ± 1°C.

### 2.7. Technological Property Measurements

#### 2.7.1. Water Binding Capacity

The water binding capacity (WBC) was determined by the Grau and Hamm method [15]. This method is based on determining the area of a wet spot on filter paper which is formed due to the slight pressure of the minced sample on it. The spot area was determined via a planimeter. The mass fraction of bound moisture was calculated according to the following formula:

(2) $$\begin{array}{c} X=\left(A-8.4\times B\right)\times 100\times A, \end{array}$$

where *X* is the mass fraction of bound water in the sample, % total moisture; *A* is the total mass of moisture in the sample, mg; and *B* is the area of the wet spot, mm^2^.

#### 2.7.2. Water Holding Capacity

Water holding capacity (WHC (%)) was determined gravimetrically according to the method described by Zhang et al. [17]. Water holding capacity (%) was calculated using the following formula:

(3) $$\begin{array}{c} \text{WHC}=\left(\frac{W_{2}-W}{W_{1}-W}\right)\times 100, \end{array}$$

where *W* is the mass of the sample, g; *W*_1_ is the mass of the sample after heating and decanting the supernatant, g; and *W*_2_ is the mass of the sample after centrifuging and removal of the resulting supernatant, g.

#### 2.7.3. Cooking Loss

Cooking loss (CL (%)) was determined using the gravimetric method, based on measuring the mass of the sample before and after heat treatment at 75°C in the air-o-steam (Rational AQ, Germany) for 10 min. Cooking loss was calculated according to the following formula:

(4) $$\begin{array}{c} \text{CL}=\left(\frac{W_{\text{rs}}-W_{\text{cs}}}{W_{\text{rs}}}\right)\times 100, \end{array}$$

where *W*_rs_ is the weight of raw sample (g) and *W*_cs_ is the weight of cooked sample (g).

### 2.8. Scanning Electron Microscopy (SEM)

The microstructures of the combined minced samples were examined by scanning in the Scanning Electron Microscope (SEM), JSM 7001F (JEOL, Japan). After freeze-drying the combined minced samples, 10 × 5 × 2 mm plates were cut from the inside of the briquettes and fixed onto the aluminium plate of the sample holder using carbon adhesive tape. The surface of the samples was covered with a layer of electrically conductive material (platinum) with a thickness of about 10 nm. The study was carried out in the registration mode for secondary electrons at a cathode accelerating voltage of 5 kV.

### 2.9. Statistical Analysis

The analyses were performed in five replicates, each of which was measured three times. The results were expressed as the mean values of the five replicates ± the standard deviation. Probability values of *p* ≤ 0.05 were taken to indicate statistical significance. The data were analysed via one-way ANOVA and the Tukey test using the free web-based software offered by Assaad et al. [18]

## 3. Results and Discussion

### 3.1. Optimization of the Combined Minced Formulation

An important condition in the development of combined products is to achieve the optimal biological value of the protein, as determined by the essential amino acid content.

The formulation of the combined mince was optimized in accordance with essential amino acid content as recommended by the WHO/FAO (World Health Organization/Food and Agriculture Organization) Expert Consultation [19]. The protein and amino acid content of beef trimming, hemp protein, and flaxseed flour (mg amino acid/100 g product) is presented in Table 2.

The balance equations were based on the experimental data obtained for the content of essential amino acids, as expressed in grams of EAAI per 100 g of protein.

The results of determining the protein content showed significant differences between hemp protein, flaxseed flour, and beef trimming protein. The protein content in the hemp protein was 56.1% (*p* < 0.05), which is close to the value obtained by Gorissen et al. [20]—51%. The crude protein content in brown and gold flax seeds is 223.25 g/kg dry matter according to Nitrayová et al. [21]. The total protein in flax seed, as found by Sammour [22], was about 20-30% of seed meal, which corresponds to the results we obtained of 39.3% (*p* < 0.05).

Hemp and flax are protein sources that provide relatively low amounts of lysine (with content of 3.55% and 3.04%, respectively), compared with beef that has a lysine content of more than 8%. This agrees with results obtained by other researchers regarding plant-based and animal-based proteins [20, 23].

Methionine and leucine content was low in flaxseed flour (3.23% and 6.06%, respectively) and did not meet the WHO/FAO requirements.

Less pronounced variability was determined between hemp, flax, and beef proteins in valine, isoleucine, and threonine content and met the WHO/FAO requirements.

The optimal ratio of minced components for which EAAI reached 100% can be determined using the SOLVER tool. Indicators of the biological value of the combined minced formulations proposed by the program were automatically calculated (Table 3).

All selected formulations have high biological value due to the necessary content of all essential amino acids in accordance with WHO/FAO requirements. The EAAI of all formulations was over 100%. Despite the content of limiting amino acids in hemp and flax, it is possible to obtain a balanced minced formulation by combining protein sources of different origin. Gorissen et al. [20] determined that plant-based proteins have relatively low essential amino acid content compared to animal-based proteins. As sources of plant-based protein, the authors studied isolates including oat, lupin, wheat, hemp, microalgae, soy, brown rice, pea, corn, and potato; milk, whey, caseinate, casein, and egg were studied as sources of animal-derived proteins. According to the results of the work, the authors concluded that a balanced combination of different plant-based proteins might provide a high-quality protein blend [20]. van Vliet et al. [20, 23] noted that the ingestion of several protein sources might provide a more balanced amino acid profile in food.

### 3.2. Physicochemical Analyses

The results of this physicochemical analysis showed that the protein content of nontreated combined minced groups was found to vary between 13.4% and 14.8%, whereas the protein content of TG-treated samples was significantly increased and determined to be between 14.1 and 15.4% (Table 4). Similar results were obtained by Uran and Yilmaz [24] in the study of burgers with the addition of various concentrations of TG and, indeed, without the addition of TG. The protein content in burgers increased from 13.40 to 14.41% with an increase in the content of TG, whilst in the control sample, the content was 13.19%. Atilgan and Kilic [25] noted that the use of different amounts of binding agents, including TG, generally did not create significant differences in protein levels among different groups of cooked ground meat.

Significant differences in the protein content in the formulations with various combinations of plant and animal raw materials were noted. The highest protein values were found for samples S-1, S-2, and S-3 (*p* < 0.05). A similar dependence was determined by Zając et al. [26], where the addition of hemp seeds to the meat loaves led to a significant increase in protein content.

The moisture content of the combined minced samples produced according to formulations 1, 2, 3, and 4 was 71.6, 72.7, 71.7, and 71.4%, respectively, while the moisture content of samples with TG (S-1+TG, S-2+TG, S-3+TG, and S-4+TG) ranged from 71.3 to 72.5%. It may be noted that the plant components did not significantly affect the moisture content of the minces. According to Atilgan and Kilic [25], the use of microbial TG in ground beef samples did not create significant differences in moisture levels compared to appropriate control groups due to the negative effect of microbial transglutaminase on the moisture level.

The fat content in our study ranged from 7.2 to 9.5% (*p* < 0.05); statistically significant increases in fat content were found in the nontreated (S-1 and S-3) and TG-treated (S-1+TG and S-3+TG) groups (*p* < 0.05). Uran and Yilmaz [24] did not notice statistically significant differences in the fat content in the control and other groups containing the enzyme in different concentrations (0.2, 0.6, 0.8, and 1%) in burger production. The fat content in the samples with the high content of plant components (S-1 and S-3) does not differ significantly but was, however, greater than in samples with a considerable content of beef trimming in formulations (S-2 and S-4). This agrees with the data obtained by Zając et al. [26], who found that the addition of hemp seed slightly increased the fat content in meat loaves.

The ash content of the combined minced samples produced according to formulations 1, 2, 3, and 4 ranged from 3.2 to 3.7%. The ash content in samples S-1 and S-1+TG and S-3 and S-3+TG was significantly increased in comparison with other samples of minced meat (*p* < 0.05); however, when TG was added to the samples of the combined minces, no significant differences were found. According to Cofrades et al. [27], the quality parameters of chicken steaks supplemented with TG showed no statistical significance compared to control samples in terms of ash content.

The results of proximate analysis obtained by Setiadi and Alisha [28] showed that the addition of transglutaminase enzyme in an animal protein source (duck meat) and vegetable protein (soy powder and texturized soy protein) did not significantly affect its nutrients.

Due to the fact that hemp, protein, and flaxseed flour contain a considerable amount of protein, lipids, and fibre, the addition of these components in an amount more than 18% led to the increase of protein, fat, and ash content in the combined minced samples.

### 3.3. Rheological Measurements

It was experimentally determined that the rheological properties of minced meat samples without TG treatment changed depending on the proportion and amount of plant raw materials. When the content of the flax flour was increased, the minimum ultimate shear stress and viscosity were observed (S-1 and S-2), while the maximum values for these indicators were achieved in samples containing about 15% hemp protein in the formulation (S-3), as well as in control samples without plant additives (*p* < 0.05).

The data obtained are related to the fact that flaxseed flour contains a considerable amount of polysaccharides, namely, mucus and pentosans, which are distinguished by a pronounced water-holding and fat-binding ability. The large amount of water and fat in the minced meat matrix leads to a weakening of the bonds between the protein molecules forming the structural network and, as a result, to a decrease in density and viscosity. Hasanvand and Rafe confirmed that flaxseed products contain anionic polysaccharides which have excellent water holding capacity due to their swelling ratio; however, they form a weak gel [29]. The authors investigated the rheological properties of rice bran protein-flaxseed gum complex coacervates and observed that coacervates showed a shear-thinning phenomenon due to the linear reduction of complex viscosity by increasing frequency [30].

**Figure 1:**
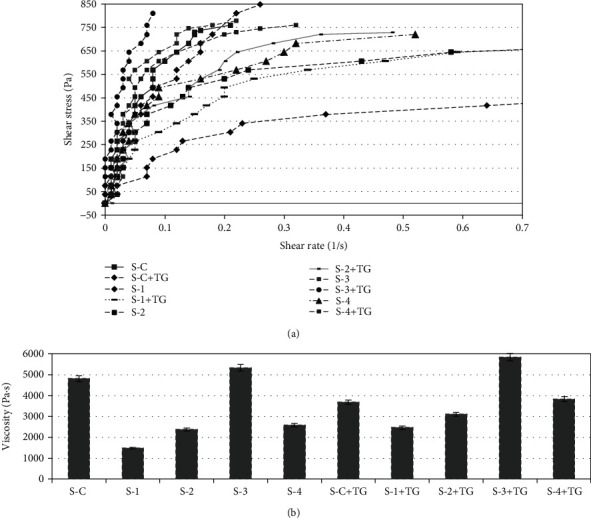
Rheological characteristics of minced meat samples: (a) dynamics of ultimate shear stress of minces depending on the rotation speed; (b) viscosity of minces depending on the rotation speed. The error bars represent the standard deviation of three separate measurements for five samples (*n* = 15). Designation of samples: S-C: the minced samples produced from beef trimming; S-C+TG: the minced samples produced from beef trimming with added TG; S-1, S-2, S-3, and S-4: the combined minced samples produced according formulations 1, 2, 3, and 4, respectively; S-1+TG, S-2+TG, S-3+TG, and S-4+TG: the combined minced samples produced according formulations 1, 2, 3, and 4, respectively, with added TG.

The ultimate shear stress and viscosity are related to the strength of intermolecular interactions in the protein-gel matrix, while the change of these indicators is related to the stability of the matrix. In the present study, the S-4 and S-C samples showed stronger intermolecular interactions and a more stable matrix. The addition of hemp might improve the formation of a homogeneous network between the meat protein and hemp protein molecules, hence producing a uniform matrix in the combined food system with improved structural strength and stability (Figures 1(a) and 1(b)).

When adding TG to the formulation, increases in the ultimate shear stress and viscosity were determined for all minced meat samples. Moreover, the TG-assisted cross-linking reaction most affected the samples with the lowest rheological properties. Thus, the ultimate shear stress in samples S-1 and S-4 increased by 44.6 and 26.7%, respectively (*p* < 0.05), while for the control sample and sample S-3, the changes were 11 and 9.9%, respectively. Therefore, the effect of TG on the formation of additional bonds between plant and animal proteins resulted in a more dense and monolithic structure (Figure 1(a)).

In the present research, the graphs for samples S-С, S-С+TG, S-3, and S-3+TG demonstrated similar dynamics and had a characteristic feature of highly concentrated gels studied at low frequency levels [31]. In general, this is a characteristic behaviour of protein-stabilized emulsions. Chattong and Apichartsrangkoon [32] noted that viscoelastic gel types with a degree of cross-link density exhibit this type of behaviour during rheological assessment. However, the replacement of more than 20% of the meat by plant substances gave the samples an excessively loose consistency and low strength characteristics that prevented the formation of semifinished products [4].

### 3.4. Texture Measurements

**Figure 2:**
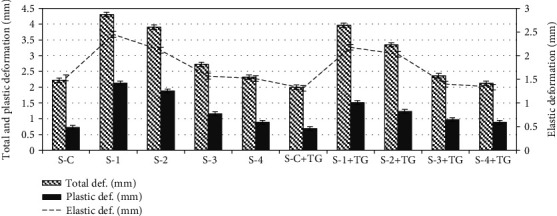
Deformation characteristics of minced meat samples. The error bars represent the standard deviation of three separate measurements for five samples (*n* = 15). Designation of samples: S-C: the minced samples produced from beef trimming; S-C+TG: the minced samples produced from beef trimming with added TG; S-1, S-2, S-3, and S-4: the combined minced samples produced according formulations 1, 2, 3, and 4, respectively; S-1+TG, S-2+TG, S-3+TG, and S-4+TG: the combined minced samples produced according formulations 1, 2, 3, and 4, respectively, with added TG.

The variation of the deformation characteristics of combined minced samples could be correlated with the results of the viscosity and critical shear stress measurements. Thus, the largest total, plastic, and elastic deformations were found in samples with the lowest shear stress and minimal viscosity (Figure 2).

The combined mince, containing more than 8% flax flour, had a more plastic structure and the lowest modulus of elasticity (total and plastic deformation for S-1 and S-2 were 3.9–4.3 and 2.1–1.9 mm, respectively). At the same time, the mince that included about 14% hemp protein (S-3) and the mince containing a more than 87% meat component (S-4) were identical to the control samples in terms of deformation and elasticity. Certainly, in TG-treated mince, the plasticity of the samples reduced while the density and elasticity increased.

### 3.5. Technological Indicator Measurements

In the combined minces, WBC was characterized by values between 76.7 and 78.8% and WHC by values from 48.5 to 52.5%, without significant variation immediately after their preparation (fermentation period 0 hours). During the first two hours of fermentation, an increase in WBC and WHC was observed for all samples without TG, whilst for samples S-1 and S-2 containing the highest concentration of flaxseed flour, the magnitudes of the indicators increased significantly by 7.3–4.9% (WBC) and 10.4–5.2% (WHC), respectively, relative to the control (*p* < 0.05) (Table 5).

For samples S-1+TG, S-2+TG, and S-3+TG, the WBC and WHC values increased significantly by 12.2–18.9% and 19.0–21.4%, respectively, after a 2 h period of TG-assisted cross-linking reaction, relative to the initial values for the same samples. The TG leads to the formation of covalent bonds between the plant and meat protein molecules that allowed water to be retained within the meat matrix under external influence, effectively and positively reducing the cooking loss. The smallest cooking losses were found for the combined minced samples characterized by the highest WBC and WHC values (S-1, S-2, and S-3). A significant decrease in CL was found for all TG-treated samples (*p* < 0.05). A similar dependence was noted by Duarte et al. [33], where the cross-links formed between the plant proteins by the action of TG greatly influenced the functional characteristics of the products, determining the technological and rheological properties of these systems such as stability, elasticity, and water adsorption. Zając et al. [26] also noted slightly decreased cooking losses in the meat loaves produced with added hemp ingredients.

Thus, TG treatment for 2 hours contributed to the formation of optimal technological properties of combined mince.

### 3.6. Scanning Electron Microscopy (SEM)

**Figure 3:**
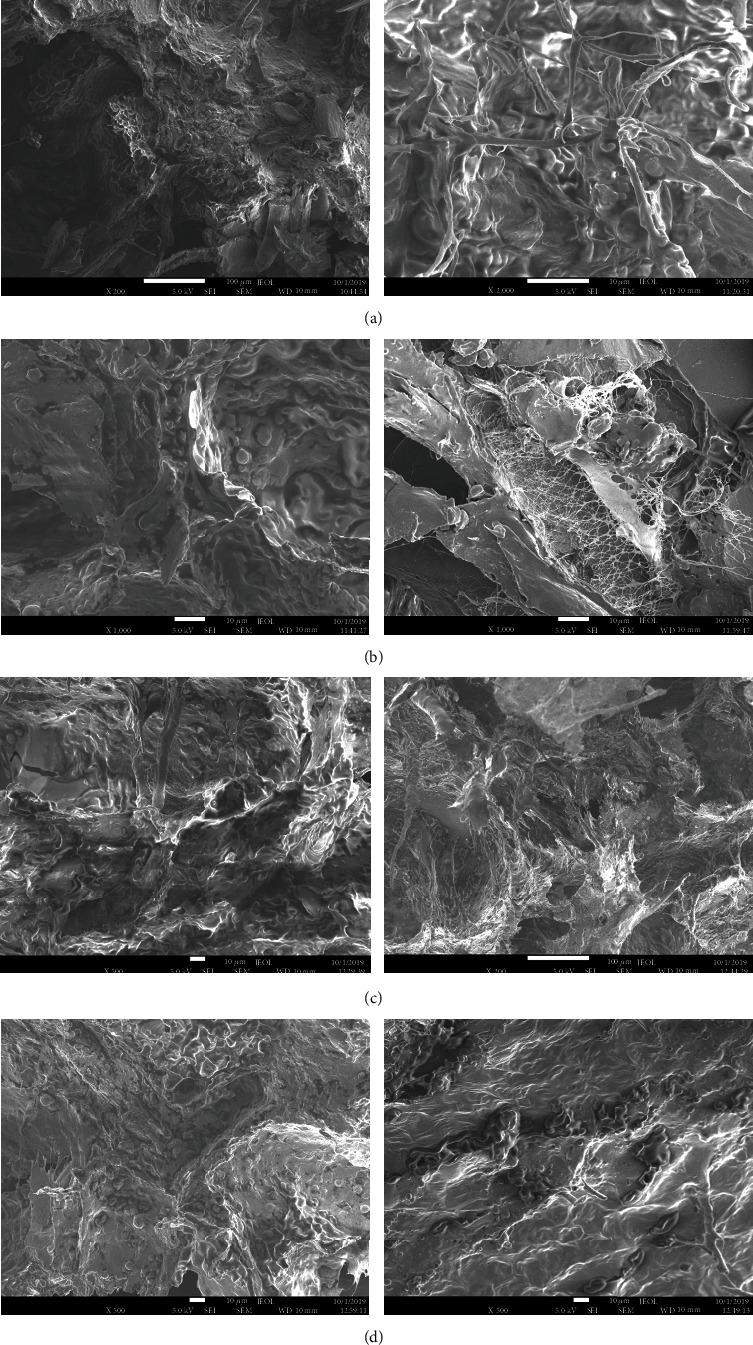
Scanning electron microscope (SEM) images of minced meat samples: (a, b, c, d) the samples produced according to formulations 1, 2, 3, and 4, respectively; (a-TG, b-TG, c-TG, d-TG) the samples produced according to formulations with added TG.

The effect of TG on the microstructural properties of combined minced samples was observed using SEM (Figure 3). The microstructures of the combined minced samples were similar in terms of the presence of certain structural elements: loose plant mass, individual plant cells, starch grains, and fragments of hemp and flax fibres, similar to that described by Jiang et al. [34], which are evenly distributed within the homogeneous meat mass.

Figures 3(c) and 3(c-TG) show SEM images of minced meat, containing about 15% hemp protein (S-3 and S-3+TG), without and with TG-treated, respectively. The observed microstructure is characterized by a pronounced homogeneity and a more uniform distribution of individual fragments, compared with the microstructure of the samples S-1, S-2, S-1+TG, and S-2+TG in Figures 3(a), 3(b), 3(a-TG), and 3(b-TG), that presents almost native flax fibres and starch grains.

The reduction in the amount of plant components in samples S-4 and S-4+TG contributed to the formation of a more compact and dens structure of minced meat, in which individual fragments are difficult to distinguish (Figures 3(d) and 3(d-TG)).

The samples of minced meat obtained without using the TG had a more open structure and greater porosity than the samples of combined mince with TG. Aksoy et al. [35] noted that the porosity and structure of freeze-dried minced meat samples depend on the temperature and drying technique. As seen in the microphotographs, the structure is more compact and denser in the combined minced samples with added TG in agreement with the results of the rheological measurements.

Li et al. [36] also reported about a more compact network of minced mixtures (pork/fish) after addition of 0.4% TG. Mojarrad et al. [37] proved that TG effects on the firmness and provided more cross-linked intermolecular gel structures at high temperatures. Gels containing MTGase supplied a stronger and denser protein network which was formed by cross-linking a network of starch and proteins [37]. The intermolecular bonds between proteins described by Uran and Yilmaz [24] as the G-L (*ε* (*γ*-glutamil)-lisil) connections in burger samples are found in combined minced samples with the addition of TG.

## 4. Conclusion

The research results have demonstrated the effectiveness of using transglutaminase in the composition of combined minced meat with beef trimming, flaxseed flour, and hemp protein for the formation of a homogeneous and dense system with the necessary technological and rheological properties. The combination of raw materials of both plant and animal origin made it possible to obtain combined minces with optimal amino acid composition of high nutritional value.

The rheological properties of minced meat samples changed depending on the proportion and amount of plant raw materials. The combined mince, containing more than 8% flax flour, had a more plastic structure and the lowest modulus of elasticity. The formulations of mince containing about 14% hemp protein or more than 87% meat component were characterized by optimal values of deformation and elasticity. When adding TG to the formulation, increase in density and elasticity, as well as growth in ultimate shear stress and viscosity, was determined for all combined minced samples.

After two hours of fermentation, an increase in water binding capacity and water holding capacity was observed for all samples. A significant decrease in cooking loss was found for all TG-treated samples. The microstructure of the TG-treated combined minced samples was more compact and dense which corresponded to the results of the rheological measurements.

## Figures and Tables

**Table 1:** The formulations of the minced meat samples.

Ingredients content (%)	Value			
	Formulation 1	Formulation 2	Formulation 3	Formulation 4
Beef trimming	78.0	82.5	79.2	87.8
Hemp protein	6.2	9.4	14.8	6.4
Flaxseed flour	15.8	8.1	6.0	5.8

**Table 2:** Essential amino acids (EAA) and protein content in beef trimming, hemp protein, and flaxseed flour.

Indicators	Beef trimming	Flaxseed flour	Hemp protein
Essential amino acids (mg/100 g sample)			
Valine	964^1^ ± 0.0775^2c^	2390 ± 0.155^b^	2940 ± 0.17^a^
Isoleucine	729 ± 0.129^c^	1970 ± 0.562^b^	2270 ± 0.187^a^
Leucine	1380 ± 0.583^c^	2380 ± 0.226^b^	4050 ± 0.336^a^
Lysine	1490 ± 0.281^b^	1190 ± 0.226^c^	1990 ± 0.228^a^
Methionine+cystine	654 ± 0.266^c^	1270 ± 0.167^b^	2770 ± 0.295^a^
Threonine	748 ± 0.259^c^	1860 ± 0.281^b^	2250 ± 0.27^a^
Tryptophan	197 ± 0.0605^c^	569 ± 0.151^a^	240 ± 0.073^b^
Phenylalanine+tyrosine	1360 ± 0.0548^c^	3800 ± 0.179^b^	4660 ± 0.464^a^
Protein (%)	17.4 ± 0.0973^c^	39.3 ± 0.0445^b^	56.1 ± 0.0864^a^

^1^Means ± ^2^standard deviation. Means in a row without a common superscript letter differ statistically (*p* < 0.05).

**Table 3:** Biological value indicators of minced meat formulations.

Content of EAA (g/100 g protein)	Value			
	Formulation 1	Formulation 2	Formulation 3	Formulation 4
Valine	5.61	5.56	5.53	5.55
Isoleucine	4.31	4.24	4.22	4.23
Leucine	7.61	7.73	7.72	7.79
Lysine	7.37	7.63	7.48	7.91
Methionine+cystine	3.75	3.83	3.90	3.80
Threonine	4.35	4.31	4.28	4.31
Tryptophan	1.14	1.09	1.05	1.10
Phenylalanine+tyrosine	8.12	7.99	7.98	7.93
EAAI (%)	115.5	115.1	114.3	115.7

EAA: essential amino acid; EAAI: essential amino acid index.

**Table 4:** The physicochemical indicators of minced meat samples.

Samples	Ash (%)	Fat (%)	Moisture (%)	Protein (%)
S-C	3.1^1^ ± 0.035^2ef^	8.8 ± 0.093^b^	68.3 ± 0.08^e^	13.1 ± 0.035^e^
S-1	3.6 ± 0.045^ab^	9.1 ± 0.073^b^	71.6 ± 0.10^cd^	13.4 ± 0.055^e^
S-2	3.2 ± 0.027^ef^	8.1 ± 0.055^c^	72.7 ± 0.095^a^	14.8 ± 0.032^b^
S-3	3.5 ± 0.035^bc^	9.1 ± 0.071^b^	71.7 ± 0.071^c^	13.9 ± 0.071^d^
S-4	3.2 ± 0.035^ef^	7.2 ± 0.071^e^	71.4 ± 0.071^cd^	14.1 ± 0.071^cd^
S-C+TG	3.2 ± 0.045^ef^	9.2 ± 0.083^b^	69.2 ± 0.10^e^	13.7 ± 0.035^ed^
S-1+TG	3.7 ± 0.035^a^	9.5 ± 0.095^a^	71.3 ± 0.089^d^	14.1 ± 0.032^cd^
S-2+TG	3.3 ± 0.042^de^	7.9 ± 0.071^cd^	72.5 ± 0.10^a^	15.4 ± 0.045^a^
S-3+TG	3.4 ± 0.035^cd^	9.5 ± 0.071^a^	71.3 ± 0.071^d^	14.2 ± 0.045^c^
S-4+TG	3.1 ± 0.047^fd^	7.6 ± 0.071^d^	72.1 ± 0.055^b^	14.7 ± 0.045^b^

^1^Means ± ^2^standard deviation. Means in a row without a common superscript letter differ statistically (*p* < 0.05). Designation of samples: S-C: the minced samples produced from beef trimming; S-C+TG: the minced samples produced from beef trimming, with added TG; S-1, S-2, S-3, and S-4: the combined minced samples produced according formulations 1, 2, 3, and 4, respectively; S-1+TG, S-2+TG, S-3+TG, and S-4+TG: the combined minced samples produced according formulations 1, 2, 3, and 4, respectively, with added TG.

**Table 5:** The technological properties of minced meat samples.

Samples	Fermentation period 0 hours			Fermentation period 2 hours		
	WBC (%)	WHC (%)	CL (%)	WBC (%)	WHC (%)	CL (%)
S-C	76.70^1^ ± 0.38^2^^b^	52.10 ± 0.33^ab^	21.52 ± 0.26^bcd^	80.84 ± 0.23^g^	54.46 ± 0.17^g^	18.72 ± 0.18^a^
S-1	78.84 ± 0.35^a^	51.64 ± 0.31^abc^	20.76 ± 0.27^de^	86.76 ± 0.27^c^	60.14 ± 0.18^bc^	15.52 ± 0.15^d^
S-2	77.52 ± 0.31^ab^	48.50 ± 0.20^d^	22.38 ± 0.4^ac^	84.80 ± 0.2^e^	57.32 ± 0.17^e^	15.34 ± 0.18^d^
S-3	77.08 ± 0.26^b^	50.30 ± 0.46^bd^	21.62 ± 0.3^bcd^	82.64 ± 0.17^f^	56.50 ± 0.19^f^	16.72 ± 0.15^c^
S-4	78.86 ± 0.35^a^	51.60 ± 0.28^abc^	19.72 ± 0.28^e^	82.20 ± 0.18^f^	55.20 ± 0.17^g^	17.56 ± 0.14^b^
S-C+TG	77.74 ± 0.33^ab^	49.50 ± 0.28^cd^	23.40 ± 0.28^a^	84.12 ± 0.14^e^	56.68 ± 0.17^ef^	16.40 ± 0.14^c^
S-1+TG	77.80 ± 0.37^ab^	51.42 ± 0.35^abc^	20.04 ± 0.25^e^	92.50 ± 0.19^a^	62.42 ± 0.13^a^	13.46 ± 0.15^f^
S-2+TG	77.66 ± 0.26^ab^	52.48 ± 0.27^ab^	21.08 ± 0.38^ce^	88.72 ± 0.18^b^	59.40 ± 0.18^c^	13.64 ± 0.12^ef^
S-3+TG	77.18 ± 0.27^b^	50.64 ± 0.33^bd^	21.52 ± 0.22^bcd^	86.60 ± 0.089^c^	60.26 ± 0.16^b^	12.96 ± 0.15^f^
S-4+TG	77.90 ± 0.45^a^	49.00 ± 0.36^d^	22.84 ± 0.21^ab^	85.70 ± 0.19^d^	58.30 ± 0.19^d^	14.24 ± 0.13^e^

^1^Means ± ^2^standard deviation. Means in a row without a common superscript letter differ statistically (*p* < 0.05). WBC: water binding capacity; WHC: water holding capacity; CL: cooking loss. Designation of samples: S-C: the minced samples produced from beef trimming; S-C+TG: the minced samples produced from beef trimming, with added TG; S-1, S-2, S-3, and S-4: the combined minced samples produced according formulations 1, 2, 3, and 4, respectively; S-1+TG, S-2+TG, S-3+TG, and S-4+TG: the combined minced samples produced according formulations 1, 2, 3, and 4, respectively, with added TG.

## Data Availability

All the experimental data used to support the findings of this study are available from the corresponding author upon request.

## Abbreviations

TG: Transglutaminase
DM: Dry matter
EAA: Essential amino acid
WBC: Water binding capacity
WHC: Water holding capacity
CL: Cooking loss.
